# Additions to the list of Finnish Bibionomorpha (Diptera, Nematocera)

**DOI:** 10.3897/BDJ.3.e5228

**Published:** 2015-06-25

**Authors:** Jukka Salmela, Kari M Kaunisto

**Affiliations:** ‡ Parks & Wildlife Finland (Metsähallitus), Rovaniemi, Finland; § Zoological Museum, Department of Biology, University of Turku, Turku, Finland

**Keywords:** Cecidomyiidae, Lestremiinae, Porricondylinae, Keroplatidae, Mycetophilidae, gall migdes, fungus gnats

## Abstract

A total of 12 gnat species are reported for the first time from Finland (3 Cecidomyiidae, 1 Keroplatidae, 8 Mycetophilidae), and the occurrence of *Macrocera nigropicea* Lundström in Finland is verified. All material was collected from the Finnish Lapland, mainly from the north boreal ecoregion. Two of the recorded species are likely to be pyrophilous, associated with forest fire sites. A photo of the ventral appendage of the gonocoxite of *Brevicornu setigerum* Zaitzev is provided for the first time. The male hypopygium of *Mycetophila haruspica* Plassmann is redescribed.

## Introduction

With over 52000 species globally, nematocerans or lower Diptera are one of the most species-rich insect groups in the world (Pape et al. 2009). Within Nematocera, the most successful terrestrial group is the Bibionomorpha, including a majority of the saproxylic, fungivorous and herbivorous species (e.g. Mycetophilidae and Cecidomyiidae, Marshall 2012). As in many other biotic groups, Fennoscandian or North European bibionomorphans are perhaps the best known on the globe, especially regarding fungus gnats (or Sciaroidea, excluding Sciaridae). Despite the taxonomic and faunistic tradition starting from the 18th century (see Kjaerandsen et al. 2007b​), species are continually added to the Nordic list (Søli and Rindal 2012, Jakovlev et al. 2014), including species new to science (e.g. Kjaerandsen et al. 2009, Salmela and Suuronen 2014, Kurina et al. 2015). No less than 915 fungus gnat species are currently known from the Nordic countries and Russian parts of Fennoscandia (Karelia and Murmansk oblast), consisting of approximately 83 % of the total European fungus gnat fauna (Kjaerandsen 2015). Nevertheless, at least 100 Fennoscandian fungus gnat species await formal naming and description (Kjaerandsen J., Polevoi A., Søli G., Salmela J., in prep.), raising the total number of species occurring in the area to around 1000. Non-herbivorous, or fungivorous and saproxylic gall midges (Cecidomyiidae) are far more poorly known than fungus gnats. In their recent monographs on the Nordic fauna, Mathias and Catrin Jaschhof (Jaschhof and Jaschhof 2009, Jaschhof and Jaschhof 2013) have described dozens of new species and have much advanced the faunistic knowledge on Lestremiinae and Porricondylinae. For example, the number of Finnish Cecidomyiidae have increased from 136 (Hackman 1980) to 356 (Jaschhof et al. 2014), mainly due to taxonomic and faunistic work performed during the last ten years. However, both Lestremiinae and Porricondylinae should still be considered as poorly known groups, and further additions to the Nordic and Finnish lists are expected.

A list of Finnish Diptera was recently published (Kahanpää and Salmela 2014). In the present paper, 12 species was added to this list (3 Cecidomyiidae, 1 Keroplatidae, 8 Mycetophilidae) and the occurrence of one keroplatid species that was erroneously deleted from the Finnish list was confirmed. Thus, the number of Finnish fungus gnats and gall midges now totals 772 and 359 species, respectively.

## Materials and methods

**Figure 1.**
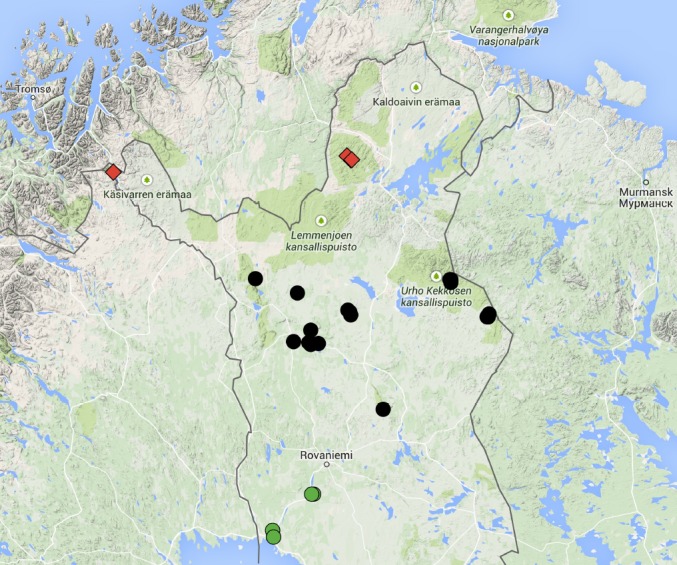
A map of Finnish Lapland and the collecting locaties of the Bibionomorpha species reported in the present paper. Green dots=middle boreal ecoregion, black dots=north boreal ecoregion, red diamonds=subalpine ecoregion. The map was created by using Google Maps.

All material reported here was collected from Finnish Lapland (Fig. 1). Lapland is an administrative area, covering a land area of ca. 100k km^2^. The SW corner of the area, close to the Baltic Sea, is middle boreal. The central part of Lapland is north boreal: coniferous forests prevail in the landscape, but there are isolated tree-less fells. Northernmost Lapland is an oroarctic area, sometimes called subalpine ecoregion. Fells and mountain birch forests prevail; isolated pine forests occur in some river valleys. The length of the thermal growing season (i.e. the number of days with the average temperature greater than + 5 C degrees after snow melt) is ca. 140-150 days in the middle boreal and ca. 105-110 days in the subalpine ecoregion (http://ilmatieteenlaitos.fi/terminen-kasvukausi, website accessed28.4.2015).

All material is deposited in the private collection of Jukka Salmela, Rovaniemi (JES). All specimens are stored in 70 % ethanol, kept in 2 ml plastic vials with screw cap and a rubber o-ring seal. Hypopygia of some specimens are kept in separate 0,5 ml microvials in glycerol.

Layer photos were taken using an Olympus E520 digital camera, attached to an Olympus SZX16 stereomicroscope. Digital photos were captured using the programmes Deep Focus 3.1 and Quick PHOTO CAMERA 2.3. Extended depth of focus images were reconstructed in the program Combine ZP.

## Taxon treatments

### Eomastix incerta

(Jaschhof, 2002)

#### Materials

- **Type status:** Other material. **Occurrence:** catalogNumber: DIPT-JS-2014-0247; recordedBy: J. Salmela; individualCount: 1; sex: male; **Location:** country: Finland; stateProvince: Lapponia kemensis pars orientalis; verbatimLocality: Savukoski, Urho Kekkonen National Park, Jaurujoki; verbatimLatitude: 68.1196; verbatimLongitude: 28.5888; verbatimCoordinateSystem: decimal degrees; verbatimSRS: WGS84; **Identification:** identifiedBy: J. Salmela; **Event:** samplingProtocol: trunk window trap; eventDate: 2014-7-1/8-5; habitat: burned, pine dominated forest, trap was set on a standing spruce; **Record Level:** institutionCode: JES
- **Type status:** Other material. **Occurrence:** catalogNumber: DIPT-JS-2014-0276; recordedBy: J. Salmela; individualCount: 10; sex: 8 male, 2 female; otherCatalogNumbers: DIPT-JS-2014-0243, DIPT-JS-2014-0341, DIPT-JS-2014-0492, DIPT-JS-2014-0493; **Location:** country: Finland; stateProvince: Lapponia kemensis pars orientalis; verbatimLocality: Savukoski, Urho Kekkonen National Park, Jaurujoki; verbatimLatitude: 68.1205; verbatimLongitude: 28.5815; verbatimCoordinateSystem: decimal degrees; verbatimSRS: WGS84; **Identification:** identifiedBy: J. Salmela; **Event:** samplingProtocol: trunk window trap; eventDate: 2009-6-4/9-16; habitat: burned, pine dominated forest, trap was set on a standing spruce; **Record Level:** institutionCode: JES

#### Distribution

**Figure 2.**
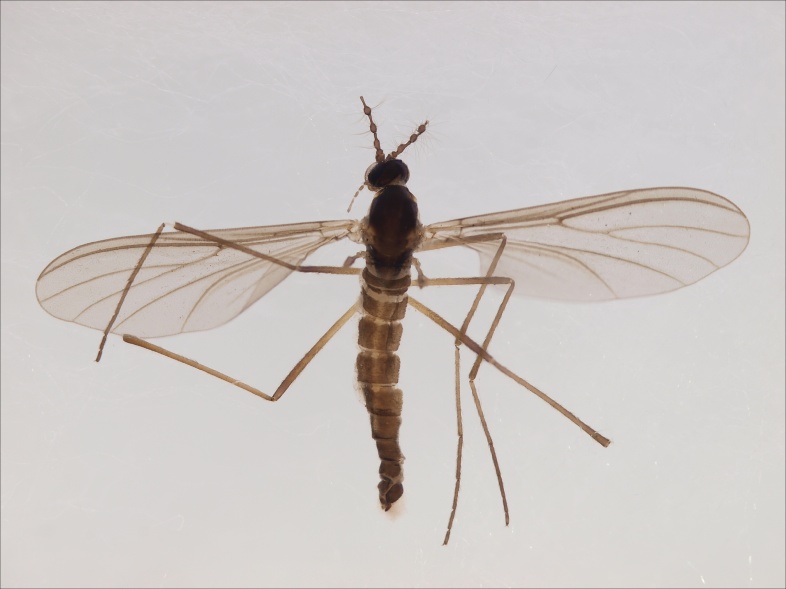
*Eomastix incerta* (Jaschhof) (Cecidomyiidae), male, DIPT-JS-2014-0492.

European. The species (Fig. 2) was described from Sweden, (Tyresta) as *Gongromastix incerta* (Jaschhof 2002), and was later transferred to a monotypic genus *Eomastix* (Jaschhof and Jaschhof 2009). The species is known from Norway and Sweden, from a single site in both countries (Jaschhof and Jaschhof 2009). The Finnish locality is in Urho Kekkonen National Park, in the north boreal zone, close to the Russian border.

#### Ecology

**Figure 3.**
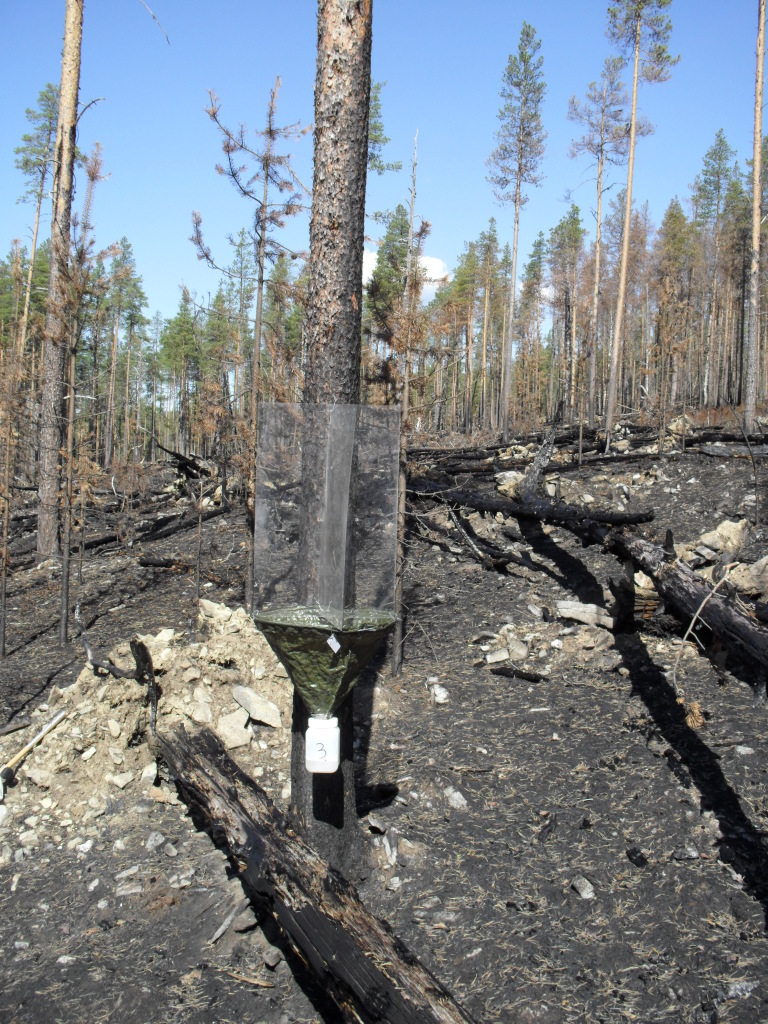
Forest fire site in Savukoski, Finnish Lapland, Urho Kekkonen National Park. Forest fire took place in 2013 and insect sampling (trunk-window traps) was performed in 2014. Two rare and poorly known ceciidomyiids, *Eomastix incerta* (Jaschhof) and *Lestremia solidaginis* (Felt), were caught. Both species are probably pyrophilous. Photo Jukka Salmela 6/2014.

Larvae of Lestreminae are perhaps mostly saproxylic (Jaschhof and Jaschhof 2009). The species is most likely to be pyrophilous, requiring or preferring forest fire areas. In Sweden, the species was collected from site that had experienced forest fire roughly one year earlier (Jaschhof and Jaschhof 2009). The Finnish collecting site is an old-growth burnt forest, dominated by pine (*Pinus sylvestris*), with scattered spruce (*Picea abies*) and birch (*Betula* sp). The forest fire site (Fig. 3) is circa 34 ha in area, and the fire was ignited by lightning in late July 2013. The species seems to have rather long flying season, from June to August.

### Lestremia solidaginis

(Felt, 1907)

#### Materials

- **Type status:** Other material. **Occurrence:** catalogNumber: DIPT-JS-2014-0343; recordedBy: J. Salmela; individualCount: 1; sex: male; **Location:** country: Finland; stateProvince: Lapponia kemensis pars orientalis; verbatimLocality: Savukoski, Urho Kekkonen National Park, Jaurujoki; verbatimLatitude: 68.1174; verbatimLongitude: 28.5814; verbatimCoordinateSystem: decimal degrees; verbatimSRS: WGS84; **Identification:** identifiedBy: J. Salmela; **Event:** samplingProtocol: trunk window trap; eventDate: 2014-8-5/9-16; habitat: burned, pine dominated forest, trap was set on a standing pine; **Record Level:** institutionCode: JES
- **Type status:** Other material. **Occurrence:** catalogNumber: DIPT-JS-2014-0350; recordedBy: J. Salmela; individualCount: 1; sex: male; **Location:** country: Finland; stateProvince: Lapponia kemensis pars orientalis; verbatimLocality: Savukoski, Urho Kekkonen National Park, Jaurujoki; verbatimLatitude: 68.1191; verbatimLongitude: 28.5844; verbatimCoordinateSystem: decimal degrees; verbatimSRS: WGS84; **Identification:** identifiedBy: J. Salmela; **Event:** samplingProtocol: trunk window trap; eventDate: 2014-8-5/9-16; habitat: burned, pine dominated forest, trap was set on a standing spruce; **Record Level:** institutionCode: JES
- **Type status:** Other material. **Occurrence:** catalogNumber: DIPT-JS-2014-0352; recordedBy: J. Salmela; individualCount: 4; sex: male; **Location:** country: Finland; stateProvince: Lapponia kemensis pars orientalis; verbatimLocality: Savukoski, Urho Kekkonen National Park, Jaurujoki; verbatimLatitude: 68.1192; verbatimLongitude: 28.5780; verbatimCoordinateSystem: decimal degrees; verbatimSRS: WGS84; **Identification:** identifiedBy: J. Salmela; **Event:** samplingProtocol: trunk window trap; eventDate: 2014-8-5/9-16; habitat: burned, pine dominated forest, trap was set on a standing pine; **Record Level:** institutionCode: JES

#### Distribution

Holarctic. The species is known to occur widely in the Nearctic region, but in the Palaearctic recorded only from southern Sweden (Jaschhof and Jaschhof 2009).

#### Ecology

Perhaps a pyrophilous species (Jaschhof and Jaschhof 2009​), a hypothesis supported by our observation. Swedish locality was a forest-fire site in Tyresta (Jaschhof 2002, Jaschhof and Jaschhof 2009). Finnish locality, identical to *Eomastix incerta*, see above.

### Porricondyla macrodon

Jaschhof, 2013

#### Materials

- **Type status:** Other material. **Occurrence:** catalogNumber: DIPT-JS-2014-0470; recordedBy: J. Salmela; individualCount: 1; sex: male; **Location:** country: Finland; stateProvince: Ostrobothnia borealis pars borealis; verbatimLocality: Keminmaa, Kallinkangas; verbatimLatitude: 65.8173; verbatimLongitude: 24.4995; verbatimCoordinateSystem: decimal degrees; verbatimSRS: WGS84; **Identification:** identifiedBy: J. Salmela; **Event:** samplingProtocol: Malaise trap; eventDate: 2014-7-28/9-23; habitat: rich fen; **Record Level:** institutionCode: JES

#### Distribution

European. The species was described recently from southern Sweden, Uppsala and Tyresta (Jaschhof and Jaschhof 2013), no other records are available. The Finnish locality is in SW Lapland, middle boreal ecoregion.

#### Ecology

The holotype specimen was collected from an "open woodland with old oaks" (Jaschhof and Jaschhof 2013). The Finnish sampling site is a rich fen, surrounded by young deciduous forest. Larvae of Porricondylinae midges are terrestrial mycelium feeders, living on detritus and dead wood (Jaschhof and Jaschhof 2013).

### Asindulum nigrum

Latreille, 1805

#### Materials

- **Type status:** Other material. **Occurrence:** catalogNumber: DIPT-JS-2014-0115; recordedBy: J. Salmela; individualCount: 1; sex: male; **Location:** country: Finland; stateProvince: Ostrobothnia borealis pars borealis; verbatimLocality: Tornio, Isonkummunjänkä Mire Conservation Area, Kusiaiskorpi; verbatimLatitude: 65.888; verbatimLongitude: 24.479; verbatimCoordinateSystem: decimal degrees; verbatimSRS: WGS84; **Identification:** identifiedBy: J. Salmela; **Event:** samplingProtocol: Malaise trap; eventDate: 2013-8-1/9-26; habitat: calcareous rich fen, rusty deposist; **Record Level:** institutionCode: JES

#### Distribution

**Figure 4.**
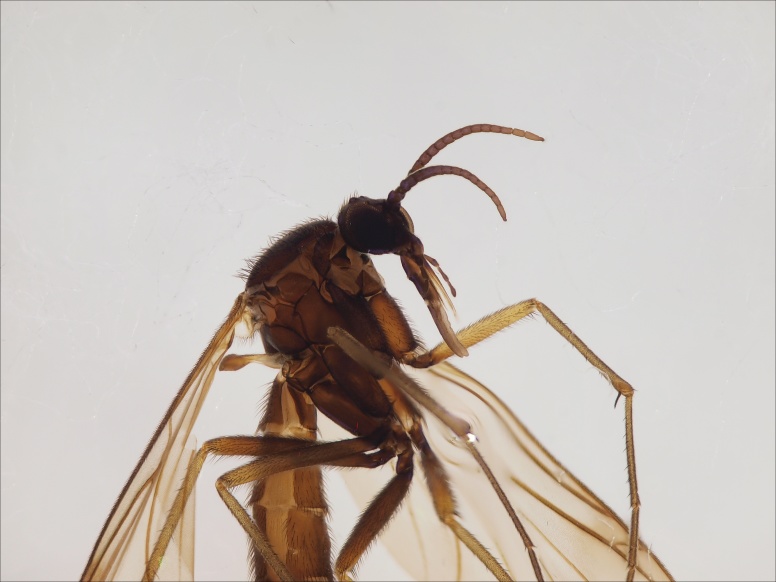
*Asindulum nigrum* Latreille (Keroplatidae), male, DIPT-JS-2014-0487. Adult flies of both sexes visit flowers and have elongated mouthparts.

European. The species (Fig. 4) was described from France (Latreille 1805) and has been later recorded from Great Britain, Central and North Europe (Matile 1975, Chandler 1991 Chandler 2004). The species seems to be rather rare throughout its range (Hedmark 2004, Chandler 1991, Ševčík and Kurina 2011b). In Sweden the species is very rare, known from the southern and central parts of the country, but it has probably vanished from four out of six of its previously occupied biogeographical provinces (Cederberg et al. 2010).

#### Ecology

Immature stages are unknown, but Orfeliini larvae are predaceous (Marshall 2012​). *Asindulum nigrum* has been collected from calcareous wetlands (Chandler 1991, Hedmark 2004) and adult flies have often been observed visiting flowers, such as Apiaceae and *Saxifraga hirculus* (Bechev 2010). Finnish collecting site is a calcareous rich fen with iron-rich seepages (for a detailed description of the habitat, see Salmela et al. 2014).

#### Conservation

The species is red-listed in Great Britain (NT, Falk and Chandler 2005) and Sweden (VU, Cederberg et al. 2010).

### Macrocera nigropicea

Lundström, 1906

#### Materials

- **Type status:** Other material. **Occurrence:** catalogNumber: MYCE-NV-2013-0035; recordedBy: J. Salmela; individualCount: 6; sex: 4 male, 2 female; **Location:** country: Finland; stateProvince: Lapponia kemensis pars occidentalis; verbatimLocality: Kittilä, Taljavaaranvuoma; verbatimLatitude: 67.577; verbatimLongitude: 25.362; verbatimCoordinateSystem: decimal degrees; verbatimSRS: WGS84; **Identification:** identifiedBy: J. Salmela; **Event:** samplingProtocol: Malaise trap; eventDate: 2007-6-25/7-24; habitat: rich fen; **Record Level:** institutionCode: JES
- **Type status:** Other material. **Occurrence:** catalogNumber: MYCE-NV-2013-0054; recordedBy: J. Salmela; individualCount: 2; sex: male; **Location:** country: Finland; stateProvince: Lapponia kemensis pars occidentalis; verbatimLocality: Kittilä, Repsuvuoma; verbatimLatitude: 67.605; verbatimLongitude: 24.964; verbatimCoordinateSystem: decimal degrees; verbatimSRS: WGS84; **Identification:** identifiedBy: J. Salmela; **Event:** samplingProtocol: Malaise trap; eventDate: 2007-6-26/7-25; habitat: rich fen; **Record Level:** institutionCode: JES
- **Type status:** Other material. **Occurrence:** catalogNumber: MYCE-NV-2013-0057; recordedBy: J. Salmela; individualCount: 2; sex: male; **Location:** country: Finland; stateProvince: Lapponia kemensis pars occidentalis; verbatimLocality: Kittilä, Silmäsvuoma; verbatimLatitude: 67.583; verbatimLongitude: 25.543; verbatimCoordinateSystem: decimal degrees; verbatimSRS: WGS84; **Identification:** identifiedBy: J. Salmela; **Event:** samplingProtocol: Malaise trap; eventDate: 2007-6-25/7-25; habitat: rich fen; **Record Level:** institutionCode: JES
- **Type status:** Other material. **Occurrence:** catalogNumber: MYCE-NV-2013-0074; recordedBy: J. Salmela; individualCount: 1; sex: male; **Location:** country: Finland; stateProvince: Lapponia kemensis pars occidentalis; verbatimLocality: Kittilä, Nunaravuoma; verbatimLatitude: 67.699; verbatimLongitude: 25.353; verbatimCoordinateSystem: decimal degrees; verbatimSRS: WGS84; **Identification:** identifiedBy: J. Salmela; **Event:** samplingProtocol: Malaise trap; eventDate: 2007-6-1/6-27; habitat: poor sedge fen; **Record Level:** institutionCode: JES
- **Type status:** Other material. **Occurrence:** catalogNumber: MYCE-NV-2013-0099; recordedBy: J. Salmela; individualCount: 1; sex: male; **Location:** country: Finland; stateProvince: Lapponia kemensis pars occidentalis; verbatimLocality: Kittilä, Kielisenpalo; verbatimLatitude: 68.021; verbatimLongitude: 25.053; verbatimCoordinateSystem: decimal degrees; verbatimSRS: WGS84; **Identification:** identifiedBy: J. Salmela; **Event:** samplingProtocol: Malaise trap; eventDate: 2007-6-26/7-27; habitat: rich spring fen; **Record Level:** institutionCode: JES
- **Type status:** Other material. **Occurrence:** catalogNumber: DIPT-JS-2014-0332; recordedBy: J. Salmela; individualCount: 5; sex: 3 female, 2 male; **Location:** country: Finland; stateProvince: Lapponia kemensis pars occidentalis; verbatimLocality: Kittilä, Akharamanvuoma; verbatimLatitude: 67.593; verbatimLongitude: 25.308; verbatimCoordinateSystem: decimal degrees; verbatimSRS: WGS84; **Identification:** identifiedBy: J. Salmela; **Event:** samplingProtocol: Malaise trap; eventDate: 2007-6-25/8-2; habitat: rich pine fen; **Record Level:** institutionCode: JES
- **Type status:** Other material. **Occurrence:** catalogNumber: DIPT-JS-2014-0414; recordedBy: J. Salmela; individualCount: 2; sex: male; **Location:** country: Finland; stateProvince: Lapponia kemensis pars orientalis; verbatimLocality: Savukoski, Törmäoja, Ahot; verbatimLatitude: 67.817; verbatimLongitude: 29.432; verbatimCoordinateSystem: decimal degrees; verbatimSRS: WGS84; **Identification:** identifiedBy: J. Salmela; **Event:** samplingProtocol: Malaise trap; eventDate: 2014-7-8/8-7; habitat: dry meadow; **Record Level:** institutionCode: JES
- **Type status:** Other material. **Occurrence:** catalogNumber: DIPT-JS-2014-0474; recordedBy: J. Salmela; individualCount: 1; sex: male; **Location:** country: Finland; stateProvince: Lapponia kemensis pars orientalis; verbatimLocality: Savukoski, Törmäoja, Ahot; verbatimLatitude: 67.821; verbatimLongitude: 29.436; verbatimCoordinateSystem: decimal degrees; verbatimSRS: WGS84; **Identification:** identifiedBy: J. Salmela; **Event:** samplingProtocol: Malaise trap; eventDate: 2014-7-8/8-7; habitat: margin of pond, surrounded by dry meadow; **Record Level:** institutionCode: JES

#### Distribution

European. The species (Fig. 5​) was described from Russia, Kola peninsula (Lundström 1906). Chandler (Chandler 1990) redescribed the species and reported it from the British Isles. Later Kjaerandsen et al. (Kjaerandsen et al. 2007a​) verified the species from Iceland and discussed the distribution and taxonomy of the species. Although Kjaerandsen et al. had studied material collected from South Finland (Ab, Karislojo [Karjalohja]; Ka, Vehkalahti; N, Esbo [Espoo], Westend, six males in total), Jakovlev (Jakovlev 2014) deleted the species from the Finnish list, assuming that the species had not been found within post WWII borders of Finland. Here we confirm the occurrence of the species in Finland, and report eight new sites from Finnish Lapland. In Fennoscandia, *M. nigropicea* is only known from Murmansk oblast and Finland.

#### Ecology

Immature stages are unknown, but *Macrocera* larvae are predaceous and mostly associated with soil and dead wood (see e.g. Falk and Chandler 2005, Ševčík and Roháček 2008). *Macrocera nigropicea* is perhaps associated with peatlands (Chandler 1990) or woodlands (Kjaerandsen et al. 2007a​). Six out of eight sites reported here are aapamires, that is, minerogenous fens with wet flarks and dry bog-level strings. Two of the sites are open, dry meadows, but not situated far from either forest or peaty pond margins. Based on our observations, *M. nigropicea* is perhaps not an obligate mire-dwelling species, but may prefer open habitats.

### Sciophila arizonensis

Zaitzev, 1982

#### Materials

- **Type status:** Other material. **Occurrence:** catalogNumber: DIPT-JS-2014-0385; recordedBy: J. Salmela; individualCount: 1; sex: male; **Location:** country: Finland; stateProvince: Ostrobothia borealis pars borealis; verbatimLocality: Kemijärvi, Pyhä-Luosto National Park, Huttuoja; verbatimLatitude: 66.9983; verbatimLongitude: 27.0265; verbatimCoordinateSystem: decimal degrees; verbatimSRS: WGS84; **Identification:** identifiedBy: J. Salmela; **Event:** samplingProtocol: Malaise trap; eventDate: 2014-8-8/9-19; habitat: rusty spring brook, pine mire, close to riparian forest; **Record Level:** institutionCode: JES

#### Distribution

**Figure 6.**
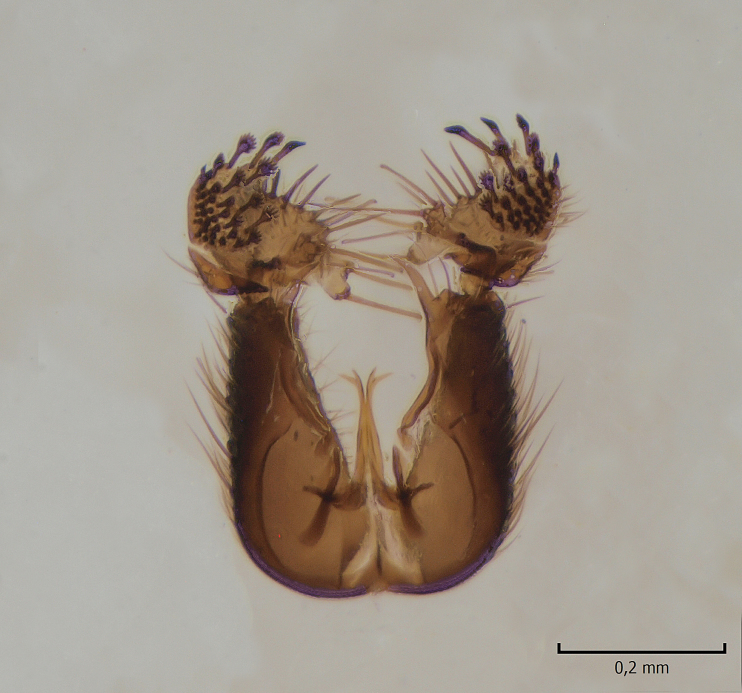
*Sciophila arizonensis* Zaitzev (Mycetophilidae), male hopygium, dorsal view, DIPT-JS-2014-0385.

Holarctic. The description of the species (Fig. 6​) was based on material collected from three Nearctic sites in Arizona, British Columbia and Ontario (Zaitzev 1982). Later the species has been recorded from the Russian Far East (Zaitzev 2006), France, Switzerland (Chandler 2004​) and the Czech Republic (Ševčík 2005). New for the Fennoscandian fauna.

#### Ecology

**Figure 7.**
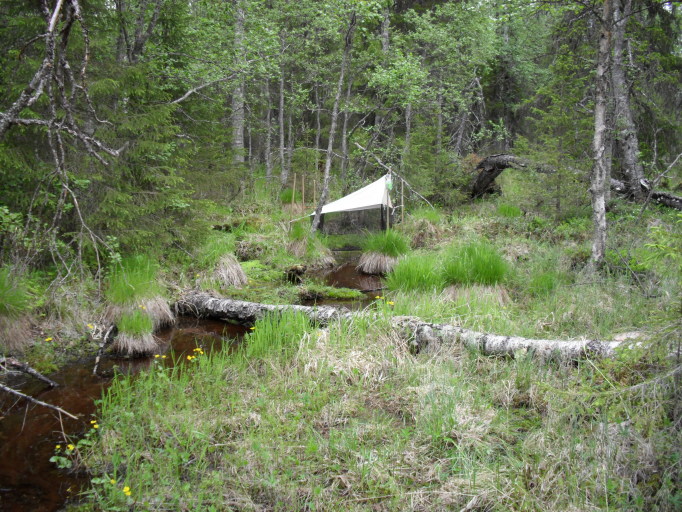
Malaise trap in Kemijärvi, Finnish Lapland, Pyhä-Luosto National Park, close to Huttuoja. The sampling site is a mixture of habitats, such as rusty spring brook, riparian forest and pine mire. Rare Holarctic fungus-gnat *Sciophila arizonensis* Zaitzev was identified from the trap material. J. Salmela 6/2014.

Immature stages are unknown, but *Sciophila* larvae are fungivorous, living on the surfaces of agaric and polyporous fungi (Ševčík 2010), rarely on Pezizales (Jakovlev 2011). Finnish locality (Fig. 7​) is a iron-rich spring-fed brook on an ecotone between a pine mire and a luxuriant riparian forest.

### Sciophila fridolini

Stackelberg, 1943

#### Materials

- **Type status:** Other material. **Occurrence:** catalogNumber: DIPT-JS-2015-0200; recordedBy: E. Rundgren; individualCount: 1; sex: male; **Location:** country: Finland; stateProvince: Lapponia inariensis; verbatimLocality: Inari, Muotkatunturi Wilderness Area, Ceavrajohoaivi; verbatimLatitude: 69.1750; verbatimLongitude: 26.2012; verbatimCoordinateSystem: decimal degrees; verbatimSRS: WGS84; **Identification:** identifiedBy: J. Salmela; **Event:** samplingProtocol: Malaise trap; eventDate: 2014-6-26/8-5; habitat: alpine headwater stream; **Record Level:** institutionCode: JES

#### Distribution

Holarctic. The species was described from Russia, Kola Peninsula (Stackelberg 1943), and has since been recorded from the British Isles (Hutson 1979), Norway (Gammelmo and Søli 2006), USA (Zaitzev 1982) and Czech Republic (Ševčík 2005). Record from East Palaearctic (Chandler 2004​) refers to Kola Peninsula, so it is not an additional area of distribution (P.J. Chandler, pers.comm.).

#### Ecology

Immature stages are unknown, but *S. fridolini* is presumably a woodland species (Falk and Chandler 2005). The Finnish collecting site is an alpine wetland along a headwater stream, characterized by *Carex* tussocks, *Viola biflora* and sparse mountain birch forest.

### Sciophila spinifera

Zaitzev, 1982

#### Materials

- **Type status:** Other material. **Occurrence:** catalogNumber: MYCE-NV-2013-0196; recordedBy: J. Salmela; individualCount: 1; sex: male; **Location:** country: Finland; stateProvince: Lapponia kemensis pars orientalis; verbatimLocality: Sodankylä, Pomokaira, Paistipuolet NE; verbatimLatitude: 67.834; verbatimLongitude: 26.270; verbatimCoordinateSystem: decimal degrees; verbatimSRS: WGS84; **Identification:** identifiedBy: J. Salmela; **Event:** samplingProtocol: Malaise trap; eventDate: 2009-6-1/6-29; habitat: intermediate rich spring fen; **Record Level:** institutionCode: JES
- **Type status:** Other material. **Occurrence:** catalogNumber: DIPT-JS-2014-0317; recordedBy: J. Salmela; individualCount: 1; sex: male; **Location:** country: Finland; stateProvince: Lapponia kemensis pars orientalis; verbatimLocality: Sodankylä, Pomokaira, Poksaselkä E; verbatimLatitude: 67.858; verbatimLongitude: 26.259; verbatimCoordinateSystem: decimal degrees; verbatimSRS: WGS84; **Identification:** identifiedBy: J. Salmela; **Event:** samplingProtocol: Malaise trap; eventDate: 2009-6-1/6-29; habitat: spring brook surrounded by old-growth spruce forest; **Record Level:** institutionCode: JES
- **Type status:** Other material. **Occurrence:** catalogNumber: DIPT-JS-2015-0238; recordedBy: E. Rundgren; individualCount: 1; sex: male; **Location:** country: Finland; stateProvince: Lapponia inariensis; verbatimLocality: Inari, Muotkatunturi Wilderness Area, Kielajoki; verbatimLatitude: 69.1464; verbatimLongitude: 26.2929; verbatimCoordinateSystem: decimal degrees; verbatimSRS: WGS84; **Identification:** identifiedBy: J. Salmela; **Event:** samplingProtocol: Malaise trap; eventDate: 2014-6-26/8-5; habitat: herb-rich swampy birch forest; **Record Level:** institutionCode: JES

#### Distribution

**Figure 8.**
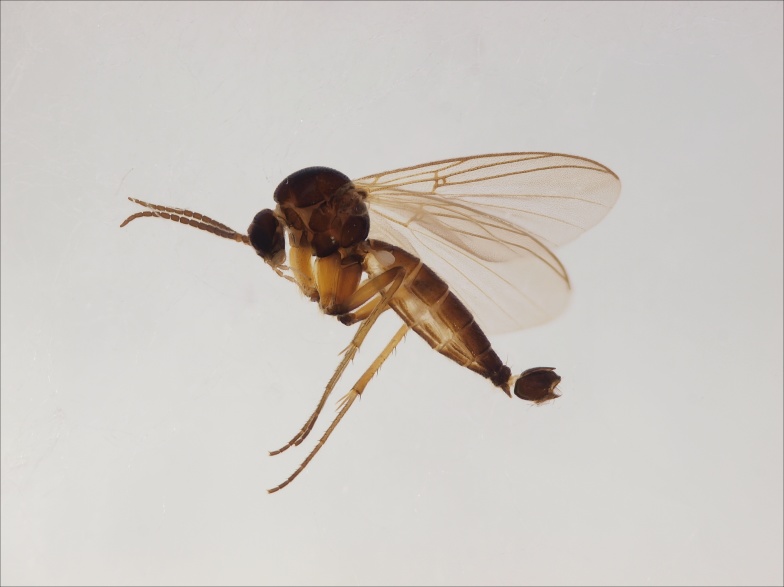
*Sciophila spinifera* Zaitzev (Mycetophilidae), male, DIPT-JS-2014-0317.

European. In his original description, Zaitzev (Zaitzev 1982) assigned *S. spinifera* (Fig. 8​) as a Finnish species, the holotype specimen was collected by Richard Frey from a place named "Opariornia". There is, however, no such place in Finland, and later the holotype was interpreted as a Swedish specimen, collected from Övertorneå (Kjaerandsen et al. 2007b​, misspelled by Zaitzev), on the Swedish side of the River Tornio between Finland and Sweden. In addition to Sweden, the species is known from southern Norway (Økland and Zaitzev 1997) and Switzerland (Chandler 1998​).

#### Ecology

Immature stages are unknown, but *S. spinifera* is presumably a forest-dwelling species (Økland and Zaitzev 1997). Finnish sampling sites are either old-growth, spruce-dominated moist forests (two sites) or a swampy birch forest.

### Allodia (Brachycampta) bohemica

Ševčík, 2004

#### Materials

- **Type status:** Other material. **Occurrence:** catalogNumber: DIPT-JS-2014-0178; recordedBy: J. Salmela; individualCount: 1; sex: male; **Location:** country: Finland; stateProvince: Ostrobothnia borealis pars borealis; verbatimLocality: Rovaniemi, Savioja; verbatimLatitude: 66.227; verbatimLongitude: 25.376; verbatimCoordinateSystem: decimal degrees; verbatimSRS: WGS84; **Identification:** identifiedBy: J. Salmela; **Event:** samplingProtocol: Malaise trap; eventDate: 2013-8-1/9-26; habitat: herb-rich forest along a headwater stream; **Record Level:** institutionCode: JES

#### Distribution

European. A rarely collected and poorly known species, reported from Czech Republic (Ševčík 2004) and Russian Karelia (Jakovlev et al. 2014). The Finnish collecting site reported here is located in SW Lapland, middle boreal ecoregion.

#### Ecology

Larvae of *Allodia* are likely to be fungivorous, see discussion in Jakovlev et al. 2014. The Finnish locality is a herb-rich riparian forest, dominated by deciduous trees.

### Brevicornu setigerum

Zaitzev, 1995

#### Materials

- **Type status:** Other material. **Occurrence:** catalogNumber: DIPT-JS-2015-0213; recordedBy: E. Rundgren; individualCount: 1; sex: male; **Location:** country: Finland; stateProvince: Lapponia inariensis; verbatimLocality: Inari, Muotkatunturi Wilderness Area, Kielajoki; verbatimLatitude: 69.1464; verbatimLongitude: 26.2929; verbatimCoordinateSystem: decimal degrees; verbatimSRS: WGS84; **Identification:** identifiedBy: J. Salmela; **Event:** samplingProtocol: Malaise trap; eventDate: 2014-6-26/8-5; habitat: herb-rich swampy birch forest; **Record Level:** institutionCode: JES
- **Type status:** Other material. **Occurrence:** catalogNumber: MYCE-JS-2013-0356; recordedBy: J. Salmela; individualCount: 1; sex: male; **Location:** country: Finland; stateProvince: Lapponia kemensis pars orientalis; verbatimLocality: Savukoski, Törmäoja, Ahot; verbatimLatitude: 67.8273; verbatimLongitude: 29.4369; verbatimCoordinateSystem: decimal degrees; verbatimSRS: WGS84; **Identification:** identifiedBy: J. Salmela; **Event:** samplingProtocol: Malaise trap; eventDate: 2013-8-7/9-19; habitat: seasonally wet meadow with large Carex-tussocs, surrounded by dry meadow; **Record Level:** institutionCode: JES
- **Type status:** Other material. **Occurrence:** catalogNumber: DIPT-JS-2014-0436; recordedBy: J. Salmela; individualCount: 1; sex: male; **Location:** country: Finland; stateProvince: Lapponia kemensis pars orientalis; verbatimLocality: Savukoski, Törmäoja, Ahot; verbatimLatitude: 67.8176; verbatimLongitude: 29.4372; verbatimCoordinateSystem: decimal degrees; verbatimSRS: WGS84; **Identification:** identifiedBy: J. Salmela; **Event:** samplingProtocol: Malaise trap; eventDate: 2014-8-8/9-19; habitat: dry meadow; **Record Level:** institutionCode: JES

#### Distribution

European. A poorly known and rarely collected Fennoscandian species (Fig. 9​). The species was described by Zaitzev (in Zaitzev and Polevoi 1995), based on a holotype male collected from Kivach Nature Reserve, Russian Karelia. Recently, the species was observed from Alta in northern Norway (Søli and Rindal 2012).

#### Ecology

Immature stages are unknown, but *Brevicornu* larvae are most likely associated with microfungi in dead wood and soil litter (Jakovlev 2011). The species is presumably a forest-dwelling fungus gnat; at least, the Norwegian sampling site was a mixed forest (Ekrem et al. 2012). The Finnish locality is a swampy birch forest in the subalpine ecoregion.

### Stigmatomeria obscura

(Winnertz, 1864)

#### Materials

- **Type status:** Other material. **Occurrence:** catalogNumber: DIPT-JS-2015-0183; recordedBy: J. Kahanpää; individualCount: 1; sex: male; **Location:** country: Finland; stateProvince: Lapponia enontekiensis; verbatimLocality: Enontekiö, Kilpisjärvi, Saana; verbatimLatitude: 69.0480; verbatimLongitude: 20.8072; verbatimCoordinateSystem: decimal degrees; verbatimSRS: WGS84; **Identification:** identifiedBy: J. Salmela; **Event:** samplingProtocol: sweep net; eventDate: 2014-7-1; habitat: road side, mountain birch forest; **Record Level:** institutionCode: JES

#### Distribution

European, but likely to have wide Palaearctic range (Kjaerandsen et al. 2007b). The species was described from Germany (Winnertz 1864), but was later tentatively considered as a junior synonym of *S. crassicornis* (Stannius) by Tuomikoski (Tuomikoski 1966). However, status of *S. obscura* as a valid species was recently reinstated (), and the species is known to occur in Germany, Sweden (Skåne) (Kjaerandsen et al. 2007b​) and Norway (Kongsvoll) (Søli and Kjaerandsen 2008). The Finnish locality is in NW Lapland, Kilpisjärvi, belonging to the Caledonian mountain range.

#### Ecology

Immature stages are unknown. The Finnish locality was a road-side in a mountain birch forest, close to Saana fell.

### Mycetophila haruspica

Plassmann, 1990

#### Materials

- **Type status:** Other material. **Occurrence:** catalogNumber: MYCE-JS-2013-0065; recordedBy: J. Salmela; individualCount: 1; sex: male; **Location:** country: Finland; stateProvince: Lapponia kemensis pars orientalis; verbatimLocality: Savukoski, Joutenoja; verbatimLatitude: 67.8213; verbatimLongitude: 29.4408; verbatimCoordinateSystem: decimal degrees; verbatimSRS: WGS84; **Identification:** identifiedBy: J. Salmela; **Event:** samplingProtocol: Malaise trap; eventDate: 2012-8-16/9-18; habitat: headwater stream, boreal forest; **Record Level:** institutionCode: JES
- **Type status:** Other material. **Occurrence:** catalogNumber: DIPT-JS-2014-0146; recordedBy: J. Salmela; individualCount: 1; sex: male; **Location:** country: Finland; stateProvince: Lapponia enontekiensis; verbatimLocality: Enontekiö, Pallas-Yllästunturi National Park, Röyninkuru; verbatimLatitude: 68.1482; verbatimLongitude: 24.0750; verbatimCoordinateSystem: decimal degrees; verbatimSRS: WGS84; **Identification:** identifiedBy: J. Salmela; **Event:** samplingProtocol: Malaise trap; eventDate: 2013-8-7/9-19; habitat: headwater stream, old-growth spruce forest; **Record Level:** institutionCode: JES
- **Type status:** Other material. **Occurrence:** catalogNumber: DIPT-JS-2014-0202; recordedBy: J. Salmela; individualCount: 1; sex: male; **Location:** country: Finland; stateProvince: Lapponia kemensis pars orientalis; verbatimLocality: Sodankylä, Pomokaira-Tenniöaapa Mire Conservation Area, Syväkuru; verbatimLatitude: 67.8731; verbatimLongitude: 26.2148; verbatimCoordinateSystem: decimal degrees; verbatimSRS: WGS84; **Identification:** identifiedBy: J. Salmela; **Event:** samplingProtocol: Malaise trap; eventDate: 2013-8-14/9-19; habitat: willow swamp with seepages, old-growth boreal forest; **Record Level:** institutionCode: JES
- **Type status:** Other material. **Occurrence:** catalogNumber: DIPT-JS-2014-0228; recordedBy: J. Salmela; individualCount: 1; sex: male; **Location:** country: Finland; stateProvince: Lapponia kemensis pars orientalis; verbatimLocality: Savukoski, Urho Kekkonen National Park, Tyyroja; verbatimLatitude: 68.1384; verbatimLongitude: 28.5723; verbatimCoordinateSystem: decimal degrees; verbatimSRS: WGS84; **Identification:** identifiedBy: J. Salmela; **Event:** samplingProtocol: Malaise trap; eventDate: 2014-6-4/7-1; habitat: spring brook, old-growth spruce forest; **Record Level:** institutionCode: JES
- **Type status:** Other material. **Occurrence:** catalogNumber: DIPT-JS-2014-0384; recordedBy: J. Salmela; individualCount: 1; sex: male; **Location:** country: Finland; stateProvince: Lapponia kemensis pars orientalis; verbatimLocality: Savukoski, Urho Kekkonen National Park, Tyyroja; verbatimLatitude: 68.1384; verbatimLongitude: 28.5723; verbatimCoordinateSystem: decimal degrees; verbatimSRS: WGS84; **Identification:** identifiedBy: J. Salmela; **Event:** samplingProtocol: Malaise trap; eventDate: 2014-8-5/9-16; habitat: spring brook, old-growth spruce forest; **Record Level:** institutionCode: JES
- **Type status:** Other material. **Occurrence:** catalogNumber: MYCE-JS-2013-0361; recordedBy: J. Salmela; individualCount: 1; sex: male; **Location:** country: Finland; stateProvince: Lapponia kemensis pars orientalis; verbatimLocality: Savukoski, Hannu Ollin vaara; verbatimLatitude: 67.8444; verbatimLongitude: 29.4689; verbatimCoordinateSystem: decimal degrees; verbatimSRS: WGS84; **Identification:** identifiedBy: J. Salmela; **Event:** samplingProtocol: Malaise trap; eventDate: 2013-8-7/9-19; habitat: spring brook, old-growth boreal forest; **Record Level:** institutionCode: JES

#### Distribution

**Figure 10.**
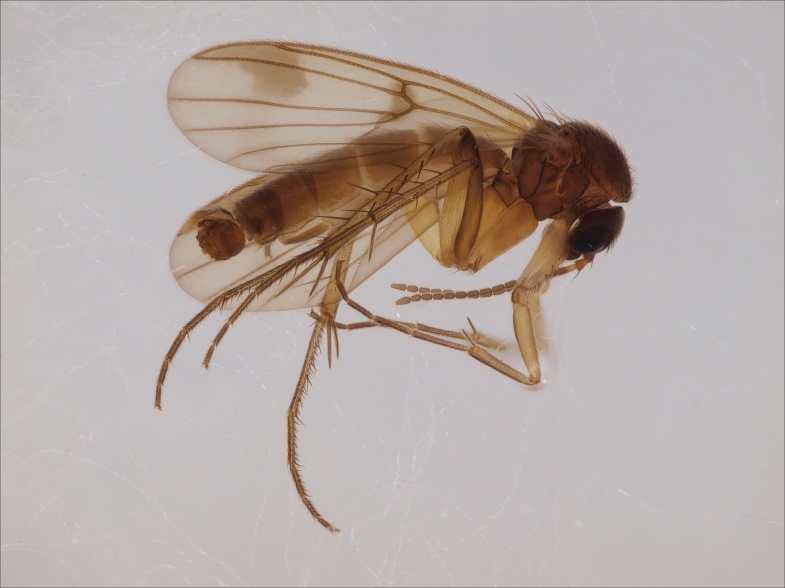
*Mycetophila haruspica* Plassmann (Mycetophilidae), male, DIPT-JS-2014-0202.

European, so far reported only from Fennoscandia. The species (Fig. 10​) was described from North Sweden (Abisko) (Plassmann 1990), and later findings are also from the northern part of the country (Kjaerandsen et al. 2007b​). Only once recorded from North Norway, Alta (Søli and Rindal 2012). Here reported from six sites in north boreal Finland.

#### Ecology

The species has been collected from both subalpine and boreal ecoregions, but detailed habitat descriptions are lacking in the literature. Finnish sampling sites are small lotic water bodies surrounded by old-growth boreal forests.

#### Taxon discussion

The original description of *M. haruspica* is rather uninformative, barely sufficient for identification purposes (Plassmann 1990, fig. 8). Proper redescription of this species is beyond the scope of this manuscript, but the morphology of the male hypopygium is reviewed here. The ventral lobe of gonostylus (vl gst, Fig. 11) has a conspicuous, helmet-like rounded lobe on dorsal margin and a small, hyaline spine next to it. Another prominent feature of vl gst is a long ventro-caudal spine, slightly bent beyond its mid-point (Fig. 11). Dorsal lobe of gonostylus (dl gst, Fig. 11) has a narrow, rounded lobe on the dorsal margin. The general shape of aedeagus is cordate, caudally tapering, apex incised and apical corners with a pair of narrow, hyaline lobes (Fig. 11c). 9th tergite is about as long as gonocoxite. Ventral margin of gonocoxites is undulating (Fig. 11d​). Based on the male hypopygium, the species is easy to separate from the other members of the vast genus *Mycetophila*.

### Mycetophila gemerensis

Ševčík & Kurina, 2011

#### Materials

- **Type status:** Other material. **Occurrence:** catalogNumber: DIPT-JS-2014-0189; recordedBy: J. Salmela; individualCount: 1; sex: male; **Location:** country: Finland; stateProvince: Ostrobothnia borealis pars borealis; verbatimLocality: Rovaniemi, Savioja; verbatimLatitude: 66.2251; verbatimLongitude: 25.3668; verbatimCoordinateSystem: decimal degrees; verbatimSRS: WGS84; **Identification:** identifiedBy: J. Salmela; **Event:** samplingProtocol: Malaise trap; eventDate: 2013-5-24/6-28; habitat: headwater stream, deciduous herb-rich forest; **Record Level:** institutionCode: JES

#### Distribution

European. The species (Fig. 12a, c​) was recently described from the Gemer region in the central part of Slovakia (Ševčík and Kurina 2011a). No other records are available. New for the Fennoscandian fauna.

#### Ecology

The holotype male was collected from a "spring area in a young spruce forest, 1230 m (above sea level)" Ševčík and Kurina 2011b). Finnish locality is a herb-rich riparian forest, dominated by deciduous trees.

#### Taxon discussion

The species is quite close to *M. lastovkai* Caspers, 1984 (Fig. 12b, d​), a species that has been in the Nordic region reported from Sweden, Norway and Denmark (Kjaerandsen 2015​). Nordic material of *M. lastovkai* should be re-examined in order to check the potential confusion with *M. gemerensis*.

## Supplementary Material

XML Treatment for Eomastix incerta

XML Treatment for Lestremia solidaginis

XML Treatment for Porricondyla macrodon

XML Treatment for Asindulum nigrum

XML Treatment for Macrocera nigropicea

XML Treatment for Sciophila arizonensis

XML Treatment for Sciophila fridolini

XML Treatment for Sciophila spinifera

XML Treatment for Allodia (Brachycampta) bohemica

XML Treatment for Brevicornu setigerum

XML Treatment for Stigmatomeria obscura

XML Treatment for Mycetophila haruspica

XML Treatment for Mycetophila gemerensis

## Figures and Tables

**Figure 5a.**
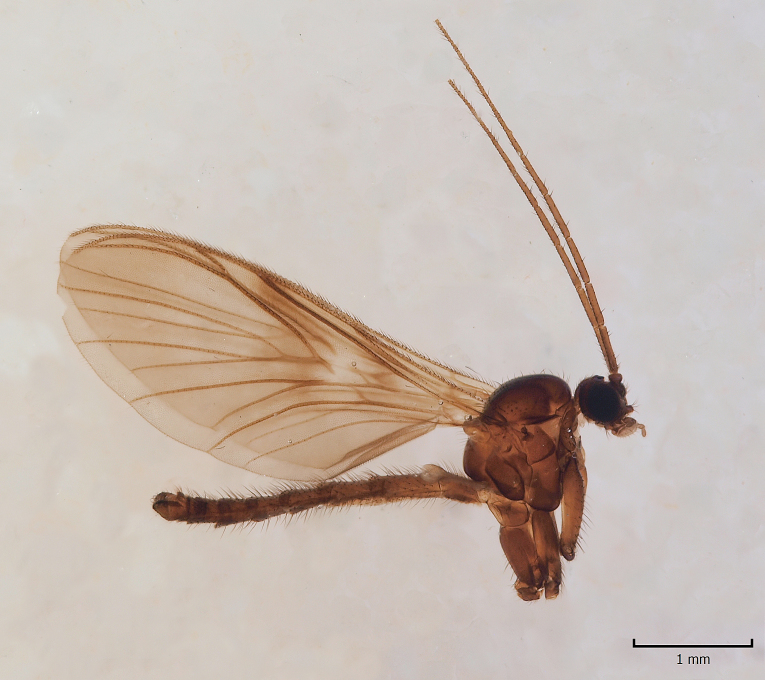
Male.

**Figure 5b.**
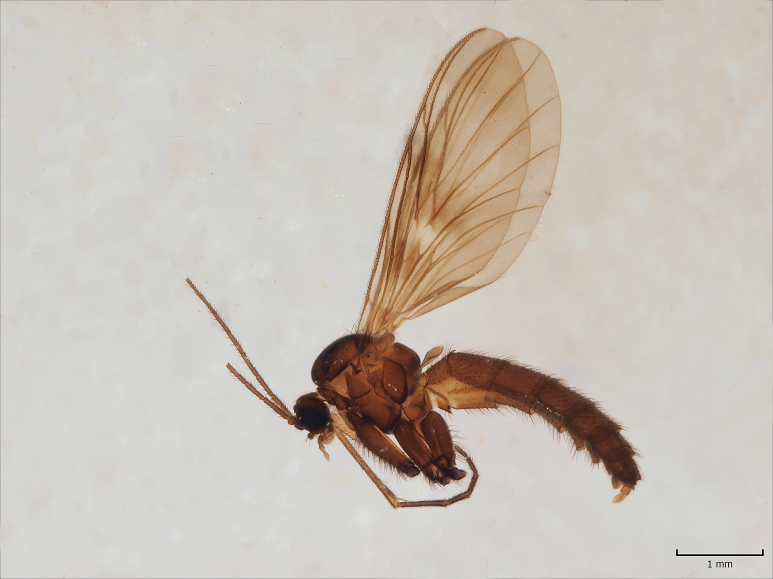
Female.

**Figure 9a.**
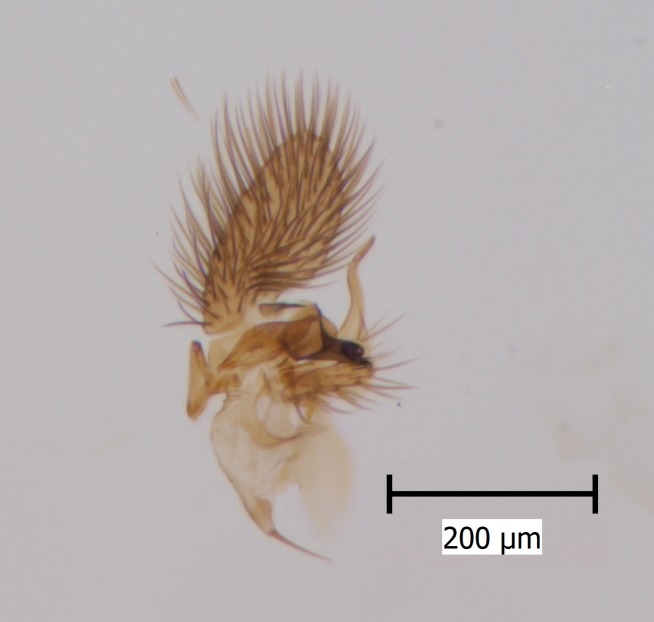
Gonostylus, lateral view.

**Figure 9b.**
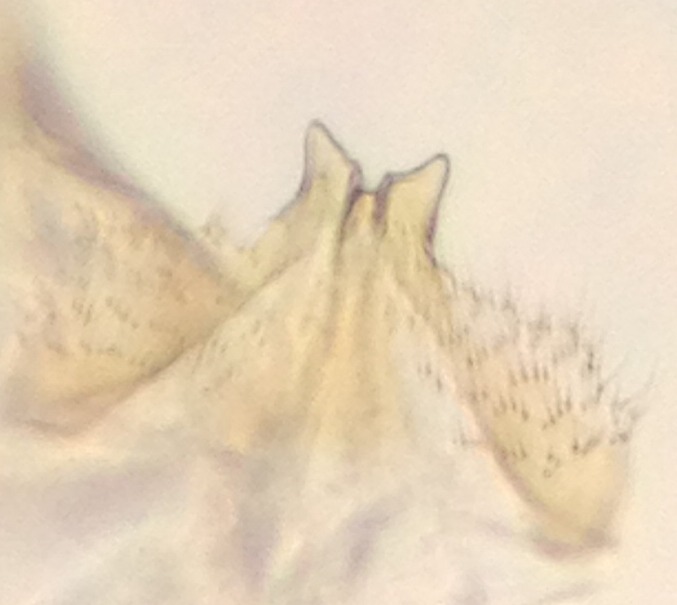
Ventral appendage of gonocoxites, ventral view; this structure has not been figured before.

**Figure 11a.**
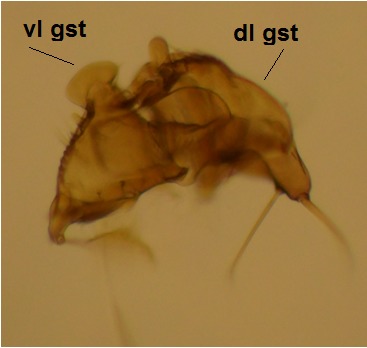
Gonostylus, lateral (inner) view. vl gst=ventral lobe of gonostylus, dl gst=dorsal lobe of gonostylus.

**Figure 11b.**
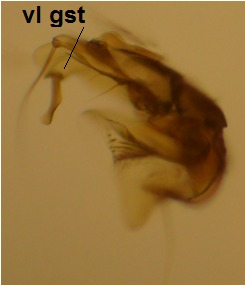
Gonostylus, ventral view.

**Figure 11c.**
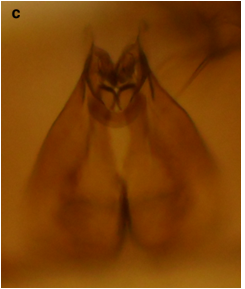
Aedeagus, dorsal view.

**Figure 11d.**
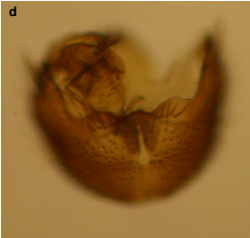
Ventral margin of gonocoxites.

**Figure 12a.**
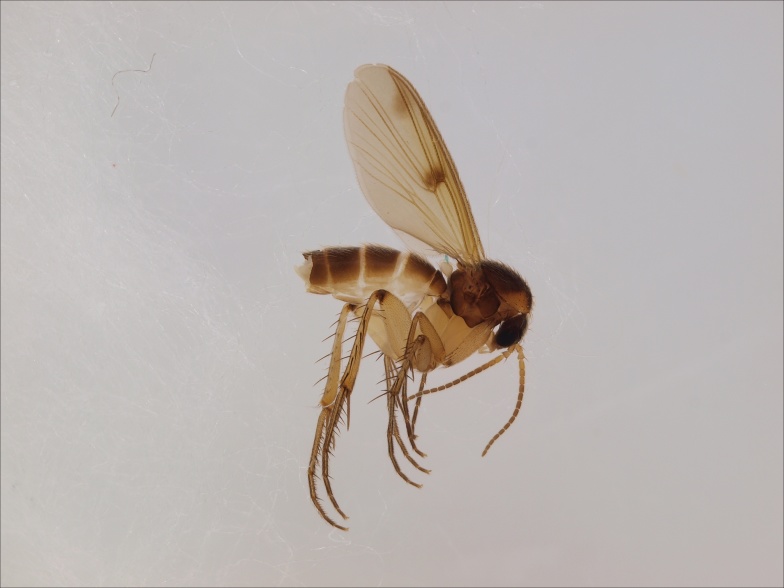
*M. gemerensis*, habitus, DIPT-JS-2014-0189.

**Figure 12b.**
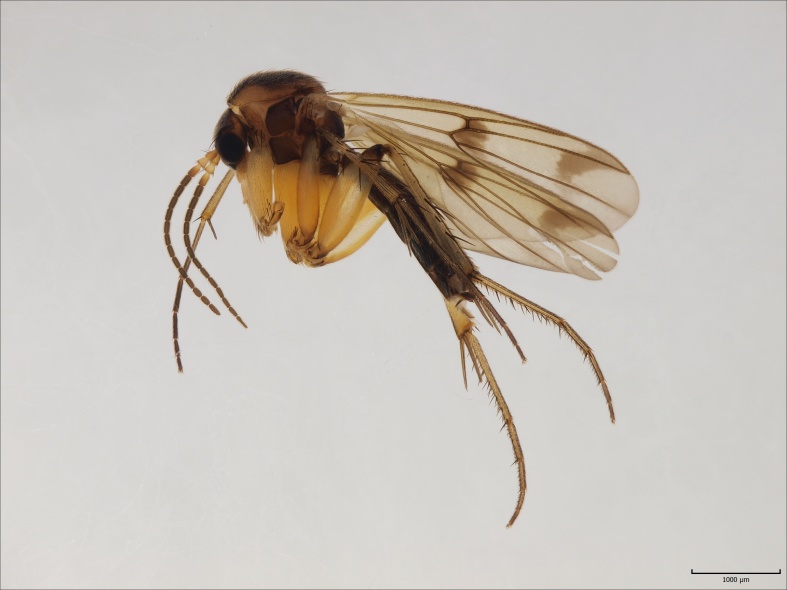
*M. lastovkai*, DIPT-JS-2014-0452, Romania, Vulcan Mt., 1420 m.a.s.l., 26.5.2014 Levente-Peter Kolcsar leg.

**Figure 12c.**
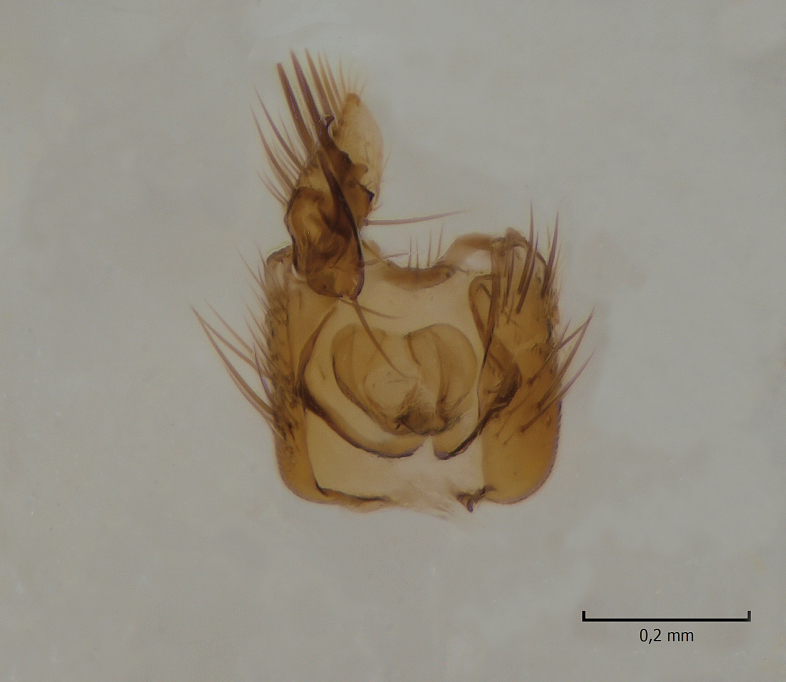
*M. gemerensis*, hypopygium, dorsal view, DIPT-JS-2014-0189.

**Figure 12d.**
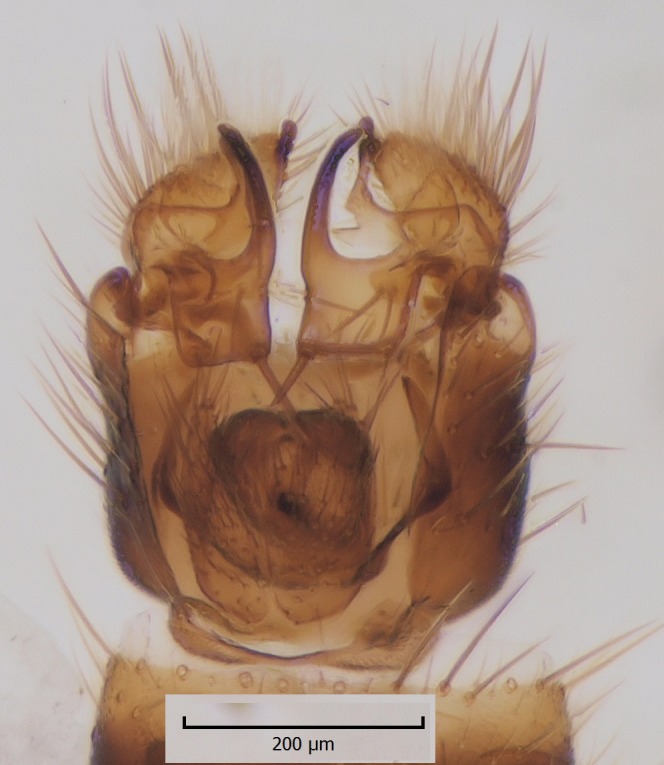
*M. lastovkai*, hypopygium, dorsal view, DIPT-JS-2014-0452.
